# Supplementary figures and images for: A k-mer-based method for the identification of phenotype-associated genomic biomarkers and predicting phenotypes of sequenced bacteria
Source: PLoS Comput Biol. 2018 Oct 22;14(10):e1006434. doi: 10.1371/journal.pcbi.1006434 (PMC6211763; doi:10.1371/journal.pcbi.1006434)

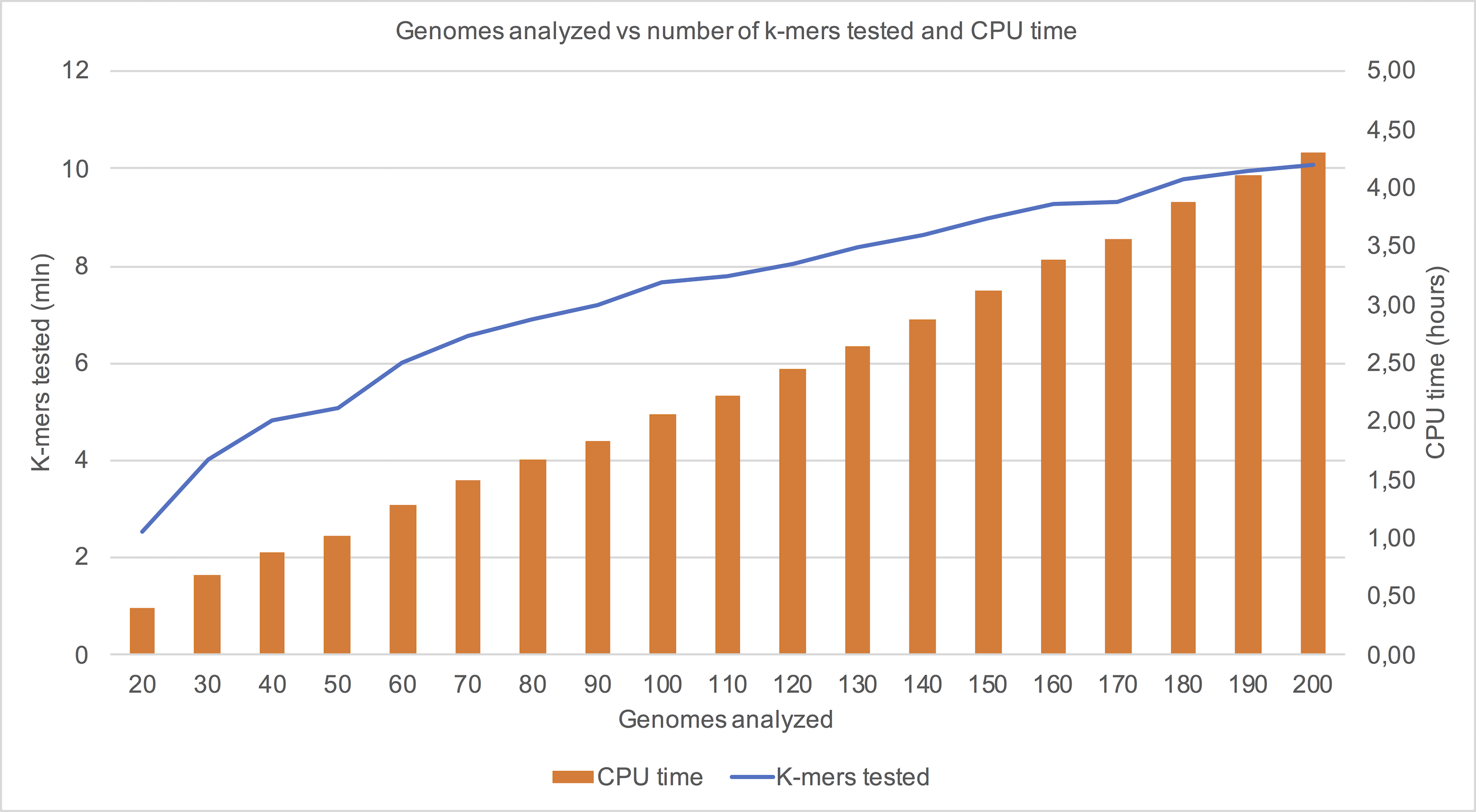

Supplement: S1 Fig — The PhenotypeSeeker CPU time depends mainly on the number of different k-mers in input genomes and on computations made with every genome. The analysis performed on our 200 P. aeruginosa genomes showed that the PhenotypeSeeker CPU time has a good linear relationship (R2 = 0.997) with the number of genomes given as input. Although the number of k-mers grows logarithmically with the number of genomes given as input, the linear relationship is because some of the computations made with every genome are more time-consuming when there are larger numbers of different k-mers present in the input genomes. (TIFF) [file pcbi.1006434.s001.tiff]

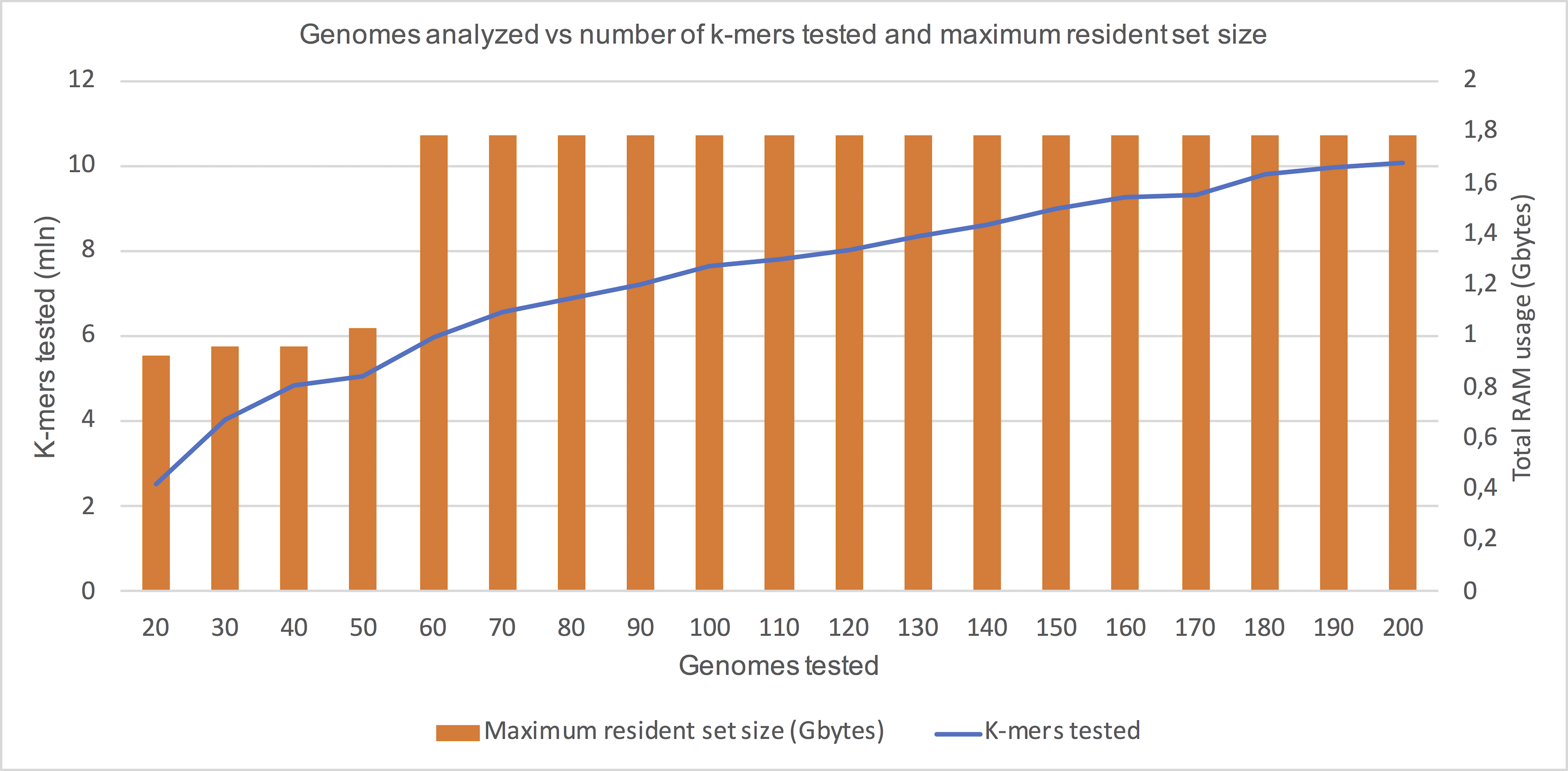

Supplement: S2 Fig — The maximum resident set size of PhenotypeSeeker increases in steps with the number of genomes that are given as the input for model training. This is due to the fact that the maximum resident set size of PhenotypeSeeker is defined by the size of the Python dictionary object into which all different k-mers and their frequencies in genomes are stored. The Python dictionary uses a hash table implementation, and the size of the hash table doubles when it is two thirds full. Therefore, when more genomes are analyzed, more different k-mers are stored into the hash table, and if a certain threshold is exceeded, the next step in the maximum resident set size is taken. However, if the regression is performed with a large number of k-mers, the regression could easily become the most memory using part of the analysis as the data matrix (k-mers x samples), read into memory, grows larger (analysis with 150, 170, 180, 190 and 200 genomes). (TIFF) [file pcbi.1006434.s002.tiff]
