# Supplementary material for: A k-mer-based method for the identification of phenotype-associated genomic biomarkers and predicting phenotypes of sequenced bacteria
Source: PLoS Comput Biol. 2018 Oct 22;14(10):e1006434. doi: 10.1371/journal.pcbi.1006434 (PMC6211763; doi:10.1371/journal.pcbi.1006434)

The confusion matrices of different classification models.

Fig S3A. The confusion matrices of classification models on contigs (N *k*-mers = 1000)

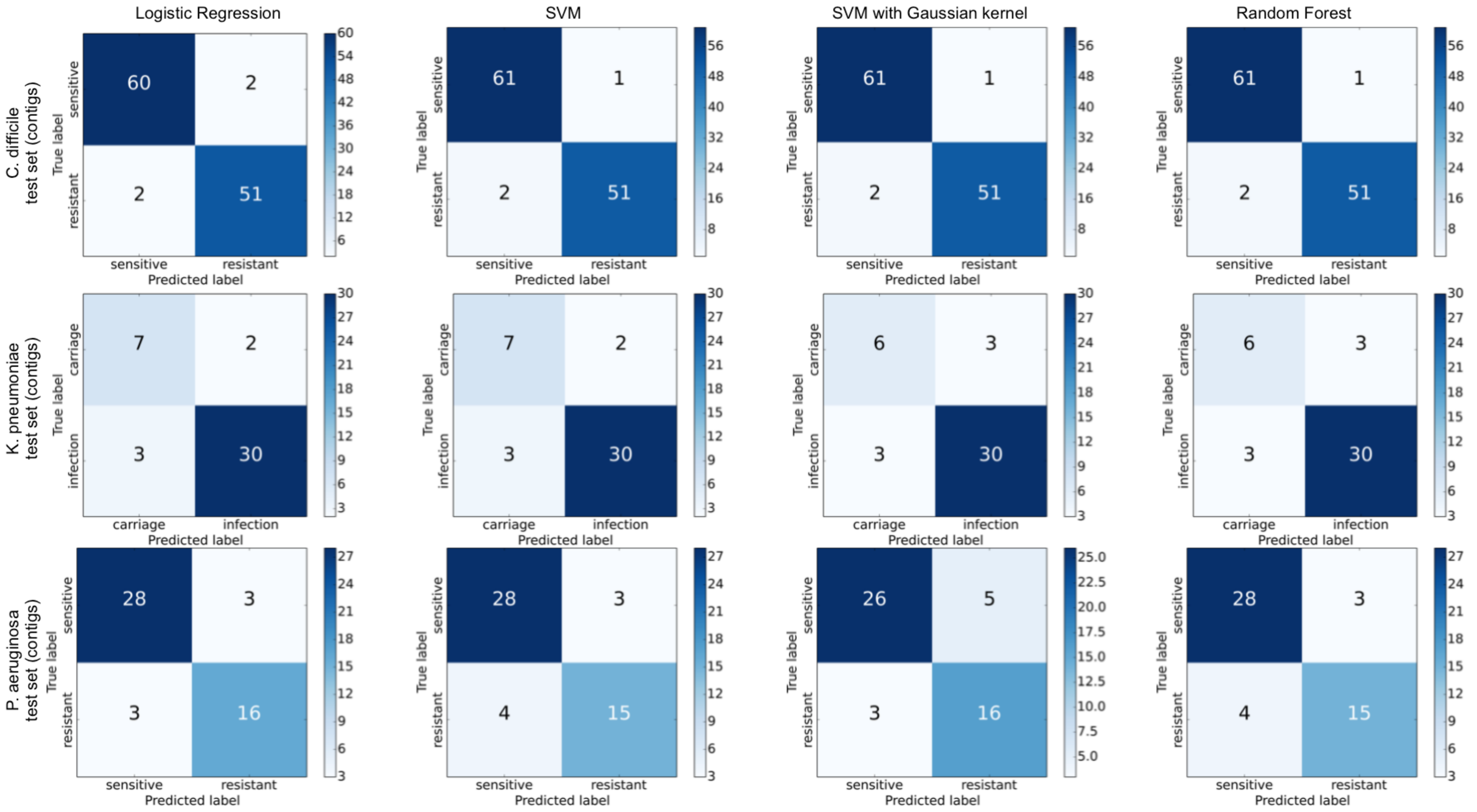

Fig S3B. The confusion matrices of classification models on reads (N *k*-mers = 1000)

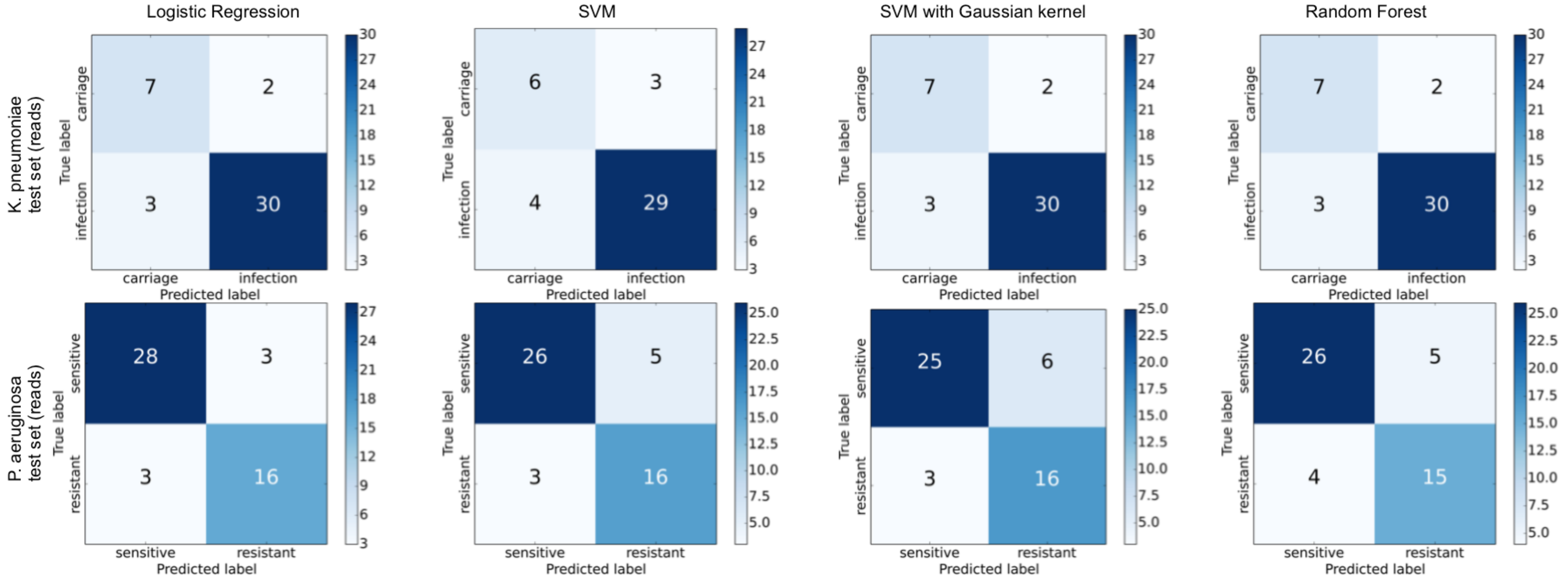

Fig S3C. The confusion matrices of classification models on contigs (N *k*-mers = 10,000)

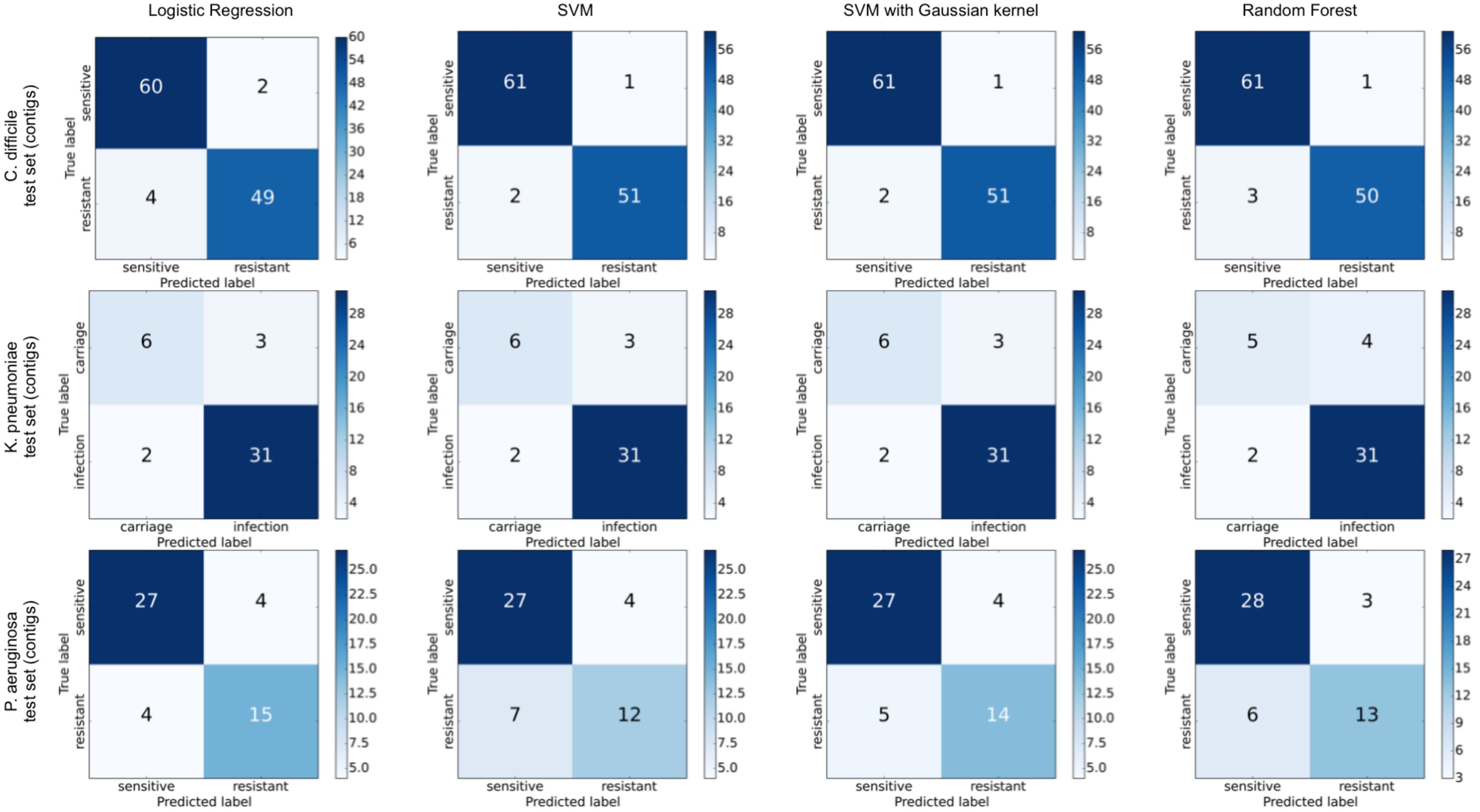

Fig S3D. The confusion matrices of classification models on reads (N *k*-mers = 10,000)

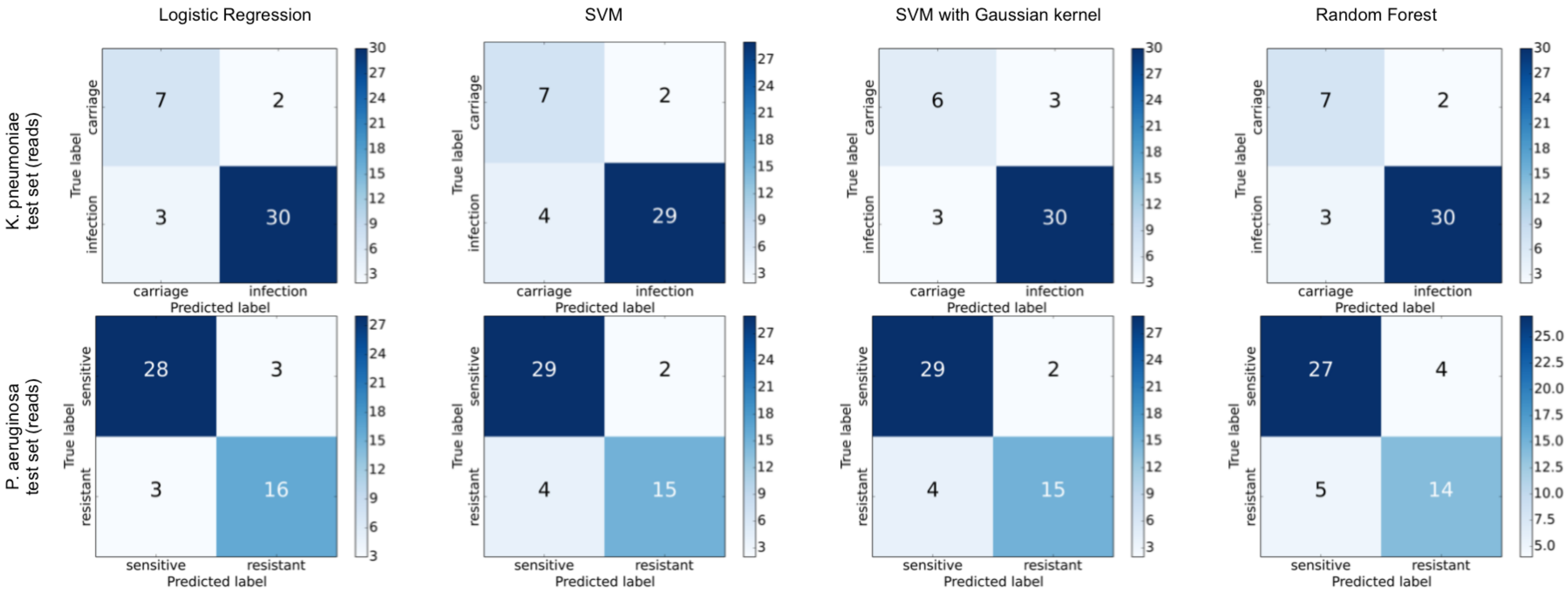

Fig S3E. The confusion matrices of classification models on contigs (N *k*-mers = 100,000)

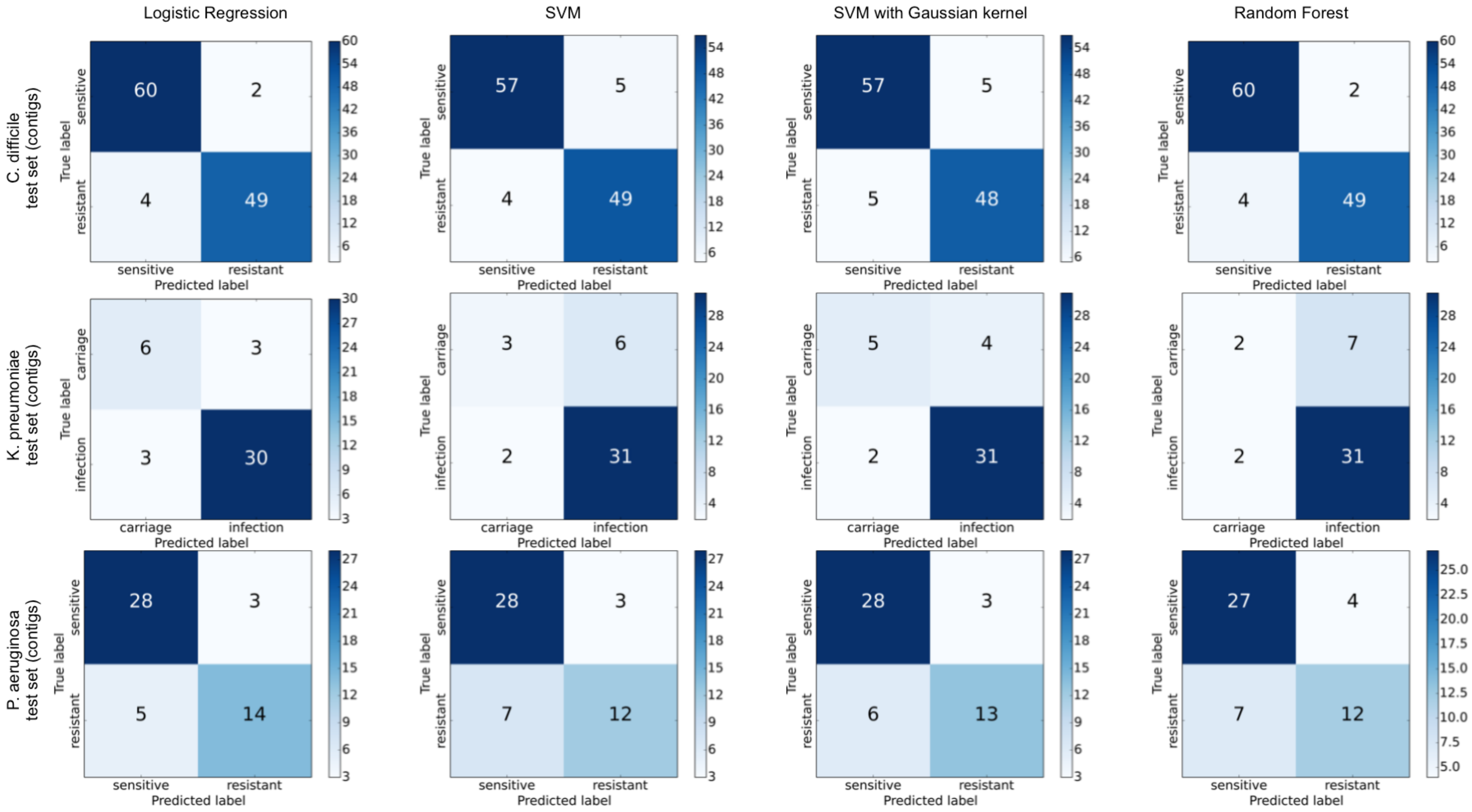

Fig S3F. The confusion matrices of classification models on contigs (N *k*-mers = 100,000)

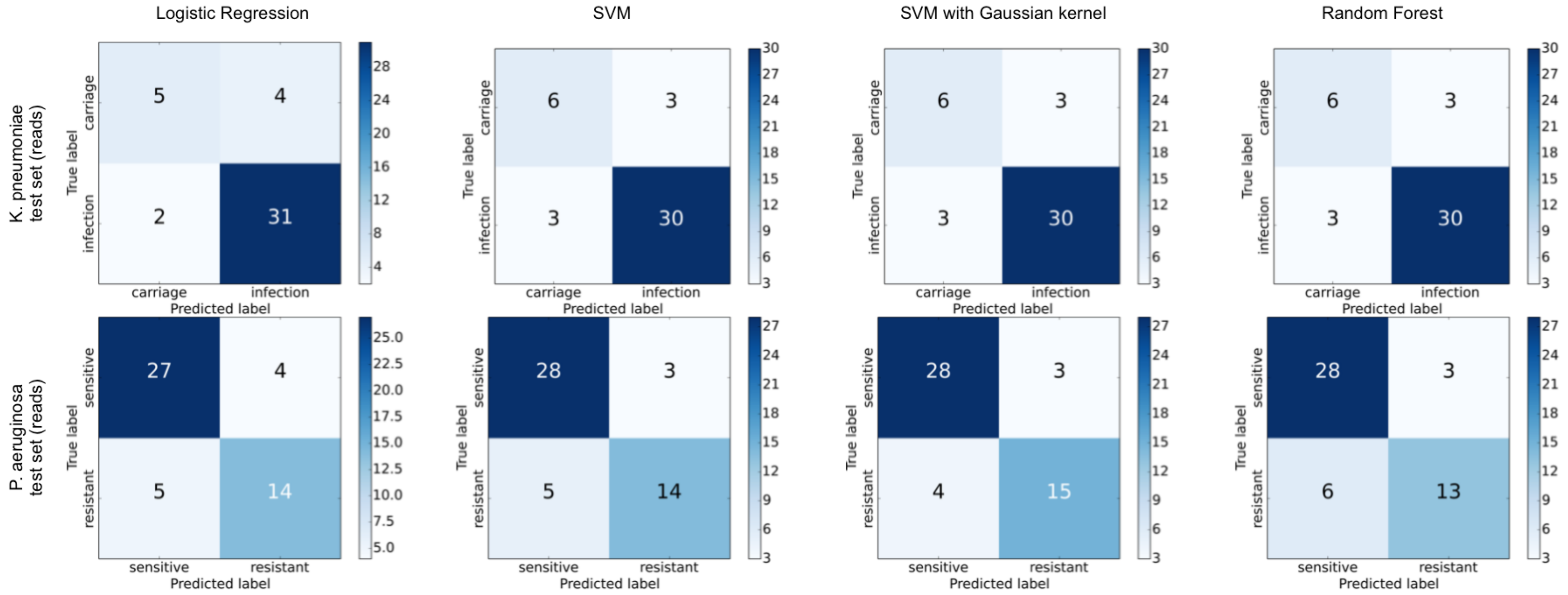

Supplement: S3 Fig — (A) The confusion matrices of classification models on contigs (N k-mers = 1,000). (B) The confusion matrices of classification models on reads (N k-mers = 1,000). (C) The confusion matrices of classification models on contigs (N k-mers = 10,000). (D) The confusion matrices of classification models on reads (N k-mers = 10,000). (E) The confusion matrices of classification models on contigs (N k-mers = 100,000). (F) The confusion matrices of classification models on reads (N k-mers = 100,000). (PDF) [file pcbi.1006434.s003.pdf]
