# Supplementary material for: A k-mer-based method for the identification of phenotype-associated genomic biomarkers and predicting phenotypes of sequenced bacteria
Source: PLoS Comput Biol. 2018 Oct 22;14(10):e1006434. doi: 10.1371/journal.pcbi.1006434 (PMC6211763; doi:10.1371/journal.pcbi.1006434)
Supplement: S1 Table — (PDF) [file pcbi.1006434.s004.pdf]

The effects of different p-value cut-offs on model perfromances on *P. aeruginosa* dataset

Table A. Results of logistic regressions performed with k-mers of length 16 and Lasso regularization. Different k-mer association p-value cut-offs were used.

Cut-off for chi-squared test p-value	1E-02	1E-03	1E-04	1E-05	1E-06	1E-07
**Total k-mers in logistic regression model**	565,742	275,243	29,642	3,569	578	102
**K-mers in model with coefficient not equal to zero**	100,948	15,978	1,562	384	81	101
**Mean accuracy on the test set**	0.76	0.8	0.82	0.78	0.84	0.8
**Sensitivity on the test set**	0.65	0.7	0.7	0.61	0.74	0.74
**Specificity on the test set**	0.85	0.89	0.93	0.93	0.93	0.85
**Avg. precision on the test set**	0.76	0.81	0.83	0.80	0.85	0.8
**Avg. recall on the test set**	0.76	0.80	0.82	0.78	0.84	0.8
**Avg. f1-score on the test set**	0.76	0.80	0.82	0.77	0.84	0.8

Table B. Results of logistic regressions performed with k-mers of length 16 and Ridge regularization. Different k-mer association p-value cut-offs were used.

Cut-off for chi-squared test p-value	1E-02	1E-03	1E-04	1E-05	1E-06	1E-07
**K-mers in logistic regression model**	565,742	275,243	29,642	3,569	578	102
**Mean accuracy on the test set**	0.8	0.78	0.78	0.78	0.84	0.8
**Sensitivity on the test set**	0.61	0.61	0.61	0.61	0.7	0.74
**Specificity on the test set**	0.96	0.93	0.93	0.93	0.89	0.85
**Avg. precision on the test set**	0.83	0.8	0.8	0.8	0.85	0.8
**Avg. recall on the test set**	0.8	0.78	0.78	0.78	0.84	0.8
**Avg. f1-score on the test set**	0.79	0.77	0.77	0.77	0.84	0.8

Table C. Results of logistic regressions performed with k-mers of length 13 and Lasso regularization. Different k-mer association p-value cut-offs were used.

Cut-off for chi-squared test p-value	1E-02	1E-03	1E-04	1E-05	1E-06	1E-07
**K-mers in logistic regression model**	291,785	127,070	17,455	2,359	385	60

**K-mers in model with coefficient not equal to zero**	11,208	62,367	822	221	91	27
**Mean accuraccy on the test set**	0.76	0.78	0.84	0.82	0.86	0.82
**Sensitivity on the test set**	0.65	0.7	0.74	0.74	0.83	0.74
**Specificity on the test set**	0.85	0.85	0.93	0.89	0.89	0.89
**Avg. precision on the test set**	0.74	0.77	0.81	0.80	0.86	0.80
**Avg. recall on the test set**	0.79	0.80	0.89	0.85	0.86	0.85
**Avg. f1-score on the test set**	0.76	0.78	0.85	0.82	0.86	0.82

Table D. Results of linear regressions performed with k-mers of length 16 and Lasso regularization. Different k-mer association p-value cut-offs were used.

**Cut-off for Welch two-sample t-test p-value**	**1E-5**	**1E-10**	**1E-20**	**1E-30**	**1E-40**
**K-mers in linear regression model**	1,551,651	1,140,036	973,402	529,634	503,636
**Mean squared error on the test subset**	6.27	5.28	5.44	7.65	9.21
**Coefficient of determination on the test subset**	0.37	0.47	0.45	0.23	0.08

Table E. Results of linear regressions performed with k-mers of length 16 (only k-mers presented in more than 10 and less than 190 strains used) and Lasso regularization. Different k-mer association p-value cut-offs were used.

**Cut-off for Welch two-sample t-test p-value**	**1E-05**	**1E-10**	**1E-15**	**1E-20**
**K-mers in linear regression model**	92,907	6,712	1,296	135
**Mean squared error on the test subset**	6.5	6.1	6.4	7.7
**Coefficient of determination on the test subset**	0.35	0.39	0.36	0.23
