# Supplementary material for: A k-mer-based method for the identification of phenotype-associated genomic biomarkers and predicting phenotypes of sequenced bacteria
Source: PLoS Comput Biol. 2018 Oct 22;14(10):e1006434. doi: 10.1371/journal.pcbi.1006434 (PMC6211763; doi:10.1371/journal.pcbi.1006434)
Supplement: S3 Table — (PDF) [file pcbi.1006434.s006.pdf]

The performance of different machine learning models on the datasets.

Table A. Performance of logistic regression models (N *k*-mers = 1,000)

Dataset		Mean accuracy	F1-score	Sensitivity	Specificity	AUC-ROC	Average precision	MCC	Cohen's kappa	Very Major Error rate	Major Error rate
**Pseudomonas aeruginosa (contigs)**	Training set	0.92	0.92	0.84	0.97	0.91	0.96	0.83	0.83	6%	2%
	Test set	0.88	0.88	0.84	0.90	0.87	0.92	0.75	0.75	6%	6%
**Pseudomonas aeruginosa (reads)**	Training set	0.95	0.95	0.90	0.99	0.94	0.99	0.90	0.90	4.0%	0.7%
	Test set	0.88	0.88	0.84	0.90	0.87	0.93	0.75	0.75	6.0%	6.0%
**Clostridium difficile (contigs)**	Training set	1.0	1.0	1.0	1.0	1.0	1.0	0.99	0.99	0.0%	0.3%
	Test set	0.97	0.97	0.96	0.97	0.97	0.97	0.93	0.93	1.7%	1.7%
**Klebsiella penumoniae (contigs)**	Training set	0.96	0.96	0.99	0.85	0.92	1.0	0.88	0.88	0.8%	3.2%
	Test set	0.88	0.88	0.91	0.78	0.84	0.97	0.66	0.66	7.1%	4.8%
**Klebsiella pneumoniae (reads)**	Training set	0.98	0.98	0.99	0.93	0.96	1.0	0.93	0.93	0.8%	1.6%
	Test set	0.88	0.88	0.91	0.78	0.84	0.98	0.66	0.66	7.1%	4.8%

Table B. Performance of logistic regression models (N *k*-mers = 10,000)

Dataset		Mean accuracy	F1-score	Sensitivity	Specificity	AUC-ROC	Average precision	MCC	Cohen's kappa	Very Major Error rate	Major Error rate
**Pseudomonas aeruginosa (contigs)**	Training set	0.90	0.90	0.83	0.95	0.89	0.95	0.79	0.78	6.7%	3.3%
	Test set	0.84	0.84	0.80	0.87	0.83	0.92	0.66	0.66	8.0%	8.0%
**Pseudomonas aeruginosa (reads)**	Training set	0.97	0.97	0.91	1.0	0.96	1.0	0.93	0.93	3.3%	0.0%
	Test set	0.88	0.88	0.84	0.90	0.87	0.90	0.75	0.75	6.0%	6.0%
**Clostridium difficile (contigs)**	Training set	0.99	0.99	0.99	1.0	0.99	1.0	0.98	0.98	0.6%	0.3%
	Test set	0.95	0.95	0.93	0.97	0.95	0.97	0.90	0.90	3.5%	1.7%
**Klebsiella penumoniae (contigs)**	Training set	1.0	1.0	1.0	1.0	1.0	1.0	1.0	1.0	0.0%	0.0%
	Test set	0.88	0.88	0.94	0.67	0.80	0.98	0.63	0.63	4.8%	7.1%
**Klebsiella pneumoniae (reads)**	Training set	1.0	1.0	1.0	1.0	1.0	1.0	1.0	1.0	0.0%	0.0%
	Test set	0.88	0.88	0.91	0.78	0.84	0.99	0.66	0.66	7.1%	4.8%

Table C. Performance of logistic regression models (N *k*-mers = 100,000)

Dataset		Mean accuracy	F1-score	Sensitivity	Specificity	AUC-ROC	Average precision	MCC	Cohen's kappa	Very Major Error rate	Major Error rate
**Pseudomonas aeruginosa (contigs)**	Training set	0.92	0.92	0.84	0.96	0.91	0.96	0.83	0.83	6.0%	2.0%
	Test set	0.84	0.84	0.74	0.90	0.82	0.89	0.66	0.65	10.0%	6.0%
**Pseudomonas aeruginosa (reads)**	Training set	1.0	1.0	1.0	1.0	1.0	1.0	1.0	1.0	0.0%	0.0%
	Test set	0.82	0.82	0.74	0.87	0.80	0.88	0.62	0.61	10.0%	8.0%
**Clostridium difficile (contigs)**	Training set	0.99	0.99	0.99	0.99	0.99	1.0	0.98	0.98	0.6%	0.6%
	Test set	0.95	0.95	0.93	0.97	0.95	0.95	0.90	0.90	3.5%	1.7%
**Klebsiella penumoniae (contigs)**	Training set	1.0	1.0	1.0	1.0	1.0	1.0	1.0	1.0	0.0%	0.0%
	Test set	0.86	0.86	0.91	0.67	0.79	0.99	0.58	0.58	7.1%	7.1%
**Klebsiella pneumoniae (reads)**	Training set	1.0	1.0	1.0	1.0	1.0	1.0	1.0	1.0	0.0%	0.0%
	Test set	0.86	0.85	0.94	0.56	0.75	0.97	0.55	0.54	4.8%	9.5%

Table D. Performance of support vector machine models (N *k*-mers = 1,000)

Dataset		Mean accuracy	F1-score	Sensitivity	Specificity	AUC-ROC	Average precision	MCC	Cohen's kappa	Very Major Error rate	Major Error rate
**Pseudomonas aeruginosa (contigs)**	Training set	0.87	0.87	0.75	0.95	0.85	0.91	0.73	0.72	9.3%	3.3%
	Test set	0.84	0.84	0.84	0.84	0.84	0.87	0.70	0.67	6.0%	10.0%
**Pseudomonas aeruginosa (reads)**	Training set	1.0	1.0	1.0	1.0	1.0	1.0	1.0	1.0	0.0%	0.0%
	Test set	0.84	0.84	0.84	0.84	0.84	0.84	0.70	0.67	6.0%	10.0%
**Clostridium difficile (contigs)**	Training set	0.99	0.99	0.99	1.0	0.99	1.0	0.99	0.99	0.3%	0.3%
	Test set	0.97	0.97	0.96	0.98	0.97	0.97	0.95	0.95	1.7%	0.9%
**Klebsiella penumoniae (contigs)**	Training set	1.0	1.0	1.0	1.0	1.0	1.0	1.0	1.0	0.0%	0.0%
	Test set	0.88	0.88	0.91	0.78	0.84	0.98	0.66	0.66	7.1%	4.8%
**Klebsiella pneumoniae (reads)**	Training set	0.99	0.99	0.99	1.0	1.0	1.0	0.98	0.98	0.8%	0.0%
	Test set	0.83	0.84	0.88	0.67	0.77	0.98	0.53	0.53	9.5%	7.1%

Table E. Performance of support vector machine models (N *k*-mers = 10,000)

Dataset		Mean accuracy	F1-score	Sensitivity	Specificity	AUC-ROC	Average precision	MCC	Cohen's kappa	Very Major Error rate	Major Error rate
**Pseudomonas aeruginosa (contigs)**	Training set	0.85	0.85	0.70	0.95	0.82	0.90	0.69	0.68	11.3%	3.3%
	Test set	0.78	0.78	0.63	0.87	0.75	0.84	0.52	0.52	14.0%	8.0%
**Pseudomonas aeruginosa (reads)**	Training set	1.0	1.0	1.0	1.0	1.0	1.0	1.0	1.0	0.0%	0.0%
	Test set	0.88	0.88	0.79	0.94	0.86	0.88	0.74	0.74	8.0%	4.0%
**Clostridium difficile (contigs)**	Training set	1.0	1.0	1.0	1.0	1.0	1.0	0.99	0.99	0.0%	0.3%
	Test set	0.97	0.97	0.96	0.98	0.97	0.97	0.95	0.95	1.7%	0.9%
**Klebsiella penumoniae (contigs)**	Training set	1.0	0.99	0.96	0.98	1.0	0.98	0.98	0.98	0.0%	0.8%
	Test set	0.88	0.88	0.94	0.67	0.80	0.98	0.63	0.63	4.8%	7.1%
**Klebsiella pneumoniae (reads)**	Training set	1.0	1.0	1.0	1.0	1.0	1.0	1.0	1.0	0.0%	0.0%
	Test set	0.86	0.86	0.88	0.78	0.83	0.99	0.61	0.61	9.5%	4.8%

Table F. Performance of support vector machine models (N *k*-mers = 100,000)

Dataset		Mean accuracy	F1-score	Sensitivity	Specificity	AUC-ROC	Average precision	MCC	Cohen's kappa	Very Major Error rate	Major Error rate
**Pseudomonas aeruginosa (contigs)**	Training set	0.95	0.95	0.91	0.98	0.95	0.99	0.90	0.90	3.3%	1.3%
	Test set	0.8	0.79	0.63	0.90	0.77	0.84	0.57	0.56	14.0%	6.0%
**Pseudomonas aeruginosa (reads)**	Training set	0.92	0.92	0.84	0.97	0.91	0.96	0.83	0.83	6.0%	2.0%
	Test set	0.84	0.84	0.74	0.90	0.82	0.90	0.66	0.65	10.0%	6.0%
**Clostridium difficile (contigs)**	Training set	1.0	1.0	1.0	1.0	1.0	1.0	1.0	1.0	0.0%	0.0%
	Test set	0.92	0.92	0.93	0.92	0.92	0.96	0.84	0.84	3.5%	4.4%
**Klebsiella penumoniae (contigs)**	Training set	0.96	0.96	1.0	0.82	0.91	1.0	0.88	0.87	0.0%	4.0%
	Test set	0.81	0.79	0.94	0.33	0.64	0.97	0.35	0.33	4.8%	14.3%
**Klebsiella pneumoniae (reads)**	Training set	0.95	0.95	0.95	0.99	0.82	1.0	0.86	0.85	0.8%	4.0%
	Test set	0.86	0.86	0.91	0.67	0.79	0.98	0.58	0.58	7.1%	7.1%

Table G. Performance of support vector machine models with Gaussian kernel function (N *k*-mers = 1,000)

Dataset		Mean accuracy	F1-score	Sensitivity	Specificity	AUC-ROC	Average precision	MCC	Cohen's kappa	Very Major Error rate	Major Error rate
**Pseudomonas aeruginosa (contigs)**	Training set	0.89	0.89	0.79	0.95	0.87	0.94	0.76	0.75	8.0%	3.3%
	Test set	0.84	0.84	0.84	0.84	0.84	0.84	0.67	0.67	6.0%	10.0%
**Pseudomonas aeruginosa (reads)**	Training set	1.0	1.0	1.0	1.0	1.0	1.0	1.0	1.0	0.0%	0.0%
	Test set	0.82	0.82	0.84	0.81	0.82	0.83	0.63	0.63	6.0%	12.0%
**Clostridium difficile (contigs)**	Training set	1.0	1.0	1.0	1.0	1.0	1.0	0.99	0.99	0.0%	0.3%
	Test set	0.97	0.97	0.96	0.98	0.97	0.97	0.95	0.95	1.7%	0.9%
**Klebsiella penumoniae (contigs)**	Training set	1.0	1.0	1.0	1.0	1.0	1.0	1.0	1.0	0.0%	0.0%
	Test set	0.86	0.86	0.91	0.67	0.79	0.99	0.58	0.58	7.1%	7.1%
**Klebsiella pneumoniae (reads)**	Training set	1.0	1.0	1.0	1.0	1.0	1.0	1.0	1.0	0.0%	0.0%
	Test set	0.88	0.88	0.91	0.78	0.84	0.98	0.66	0.66	7.1%	4.8%

Table H. Performance of support vector machine models with Gaussian kernel function (N *k*-mers = 10,000)

Dataset		Mean accuracy	F1-score	Sensitivity	Specificity	AUC-ROC	Average precision	MCC	Cohen's kappa	Very Major Error rate	Major Error rate
**Pseudomonas aeruginosa (contigs)**	Training set	0.99	0.99	0.98	0.99	0.99	1.0	0.97	0.97	0.7%	0.7%
	Test set	0.82	0.82	0.74	0.87	0.80	0.86	0.62	0.61	10.0%	8.0%
**Pseudomonas aeruginosa (reads)**	Training set	0.99	0.99	0.98	1.0	0.99	1.0	0.99	0.99	0.67%	0.0%
	Test set	0.88	0.88	0.79	0.94	0.86	0.88	0.74	0.74	8.0%	4.0%
**Clostridium difficile (contigs)**	Training set	1.0	1.0	1.0	1.0	1.0	1.0	1.0	1.0	0.0%	0.0%
	Test set	0.97	0.97	0.96	0.98	0.97	0.97	0.95	0.95	1.7%	0.9%
**Klebsiella penumoniae (contigs)**	Training set	1.0	1.0	1.0	1.0	1.0	1.0	1.0	1.0	0.0%	0.0%
	Test set	0.88	0.88	0.94	0.67	0.80	0.98	0.63	0.63	4.8%	7.1%
**Klebsiella pneumoniae (reads)**	Training set	1.0	1.0	1.0	1.0	1.0	1.0	1.0	1.0	0.0%	0.0%
	Test set	0.86	0.86	0.91	0.67	0.79	0.95	0.58	0.58	7.1%	7.1%

Table I. Performance of support vector machine models with Gaussian kernel function (N *k*-mers = 100,000)

Dataset		Mean accuracy	F1-score	Sensitivity	Specificity	AUC-ROC	Average precision	MCC	Cohen's kappa	Very Major Error rate	Major Error rate
**Pseudomonas aeruginosa (contigs)**	Training set	0.97	0.97	0.93	0.99	0.96	1.0	0.93	0.93	2.7%	0.7%
	Test set	0.82	0.82	0.68	0.90	0.79	0.87	0.61	0.61	12.0%	6.0%
**Pseudomonas aeruginosa (reads)**	Training set	1.0	1.0	1.0	1.0	1.0	1.0	1.0	1.0	0.0%	0.0%
	Test set	0.86	0.86	0.80	0.90	0.85	0.88	0.70	0.70	8.0%	6.0%
**Clostridium difficile (contigs)**	Training set	1.0	1.0	1.0	1.0	1.0	1.0	1.0	1.0	0.0%	0.0%
	Test set	0.91	0.91	0.91	0.92	0.91	0.95	0.83	0.83	4.4%	4.4%
**Klebsiella penumoniae (contigs)**	Training set	1.0	1.0	1.0	1.0	1.0	1.0	1.0	1.0	0.0%	0.0%
	Test set	0.86	0.85	0.94	0.56	0.75	0.98	0.55	0.54	4.8%	9.5%
**Klebsiella pneumoniae (reads)**	Training set	1.0	1.0	1.0	1.0	1.0	1.0	1.0	1.0	0.0%	0.0%
	Test set	0.86	0.86	0.91	0.67	0.79	0.99	0.58	0.58	7.1%	7.1%

Table J. Performance of random forest models (N *k*-mers = 1,000)

Dataset		Mean accuracy	F1-score	Sensitivity	Specificity	AUC-ROC	Average precision	MCC	Cohen's kappa	Very Major Error rate	Major Error rate
**Pseudomonas aeruginosa (contigs)**	Training set	0.99	0.99	0.98	0.99	0.99	0.99	0.97	0.97	0.7%	0.7%
	Test set	0.86	0.86	0.79	0.90	0.85	0.90	0.70	0.70	8.0%	6.0%
**Pseudomonas aeruginosa (reads)**	Training set	1.0	1.0	1.0	1.0	1.0	1.0	1.0	1.0	0.0%	0.0%
	Test set	0.82	0.82	0.80	0.84	0.81	0.85	0.62	0.62	8.0%	10.0%
**Clostridium difficile (contigs)**	Training set	1.0	1.0	1.0	1.0	1.0	1.0	1.0	1.0	0.0%	0.0%
	Test set	0.97	0.97	0.96	0.98	0.97	0.98	0.95	0.95	1.7%	0.9%
**Klebsiella penumoniae (contigs)**	Training set	1.0	1.0	1.0	1.0	1.0	1.0	1.0	1.0	0.0%	0.0%
	Test set	0.86	0.86	0.91	0.67	0.79	0.92	0.58	0.58	7.1%	7.1%
**Klebsiella pneumoniae (reads)**	Training set	1.0	1.0	1.0	1.0	1.0	1.0	1.0	1.0	0.0%	0.0%
	Test set	0.88	0.88	0.91	0.78	0.84	0.98	0.67	0.67	7.1%	4.8%

Table K. Performance of random forest models (N *k*-mers = 10,000)

Dataset		Mean accuracy	F1-score	Sensitivity	Specificity	AUC-ROC	Average precision	MCC	Cohen's kappa	Very Major Error rate	Major Error rate
**Pseudomonas aeruginosa (contigs)**	Training set	0.99	0.99	1.0	0.99	0.99	1.0	0.99	0.99	0.0%	0.67%
	Test set	0.82	0.82	0.68	0.90	0.79	0.86	0.61	0.61	12.0%	6.0%
**Pseudomonas aeruginosa (reads)**	Training set	1.0	1.0	1.0	1.0	1.0	1.0	1.0	1.0	0.0%	0.0%
	Test set	0.82	0.82	0.74	0.87	0.80	0.88	0.61	0.61	10.0%	8.0%
**Clostridium difficile (contigs)**	Training set	1.0	1.0	1.0	1.0	1.0	1.0	1.0	1.0	0.0%	0.0%
	Test set	0.97	0.97	0.94	0.98	0.96	0.98	0.93	0.93	2.6%	0.9%
**Klebsiella penumoniae (contigs)**	Training set	1.0	1.0	1.0	1.0	1.0	1.0	1.0	1.0	0.0%	0.0%
	Test set	0.86	0.85	0.94	0.56	0.75	0.97	0.55	0.54	4.8%	9.5%
**Klebsiella pneumoniae (reads)**	Training set	1.0	1.0	1.0	1.0	1.0	1.0	1.0	1.0	0.0%	0.0%
	Test set	0.88	0.88	0.91	0.78	0.84	0.99	0.67	0.67	7.1%	4.8%

Table L. Performance of random forest models (N *k*-mers = 100,000)

Dataset		Mean accuracy	F1-score	Sensitivity	Specificity	AUC-ROC	Average precision	MCC	Cohen's kappa	Very Major Error rate	Major Error rate
**Pseudomonas aeruginosa (contigs)**	Training set	1.0	1.0	1.0	1.0	1.0	1.0	1.0	1.0	0.0%	0.0%
	Test set	0.78	0.78	0.63	0.87	0.75	0.86	0.52	0.52	14.0%	8.0%
**Pseudomonas aeruginosa (reads)**	Training set	1.0	1.0	1.0	1.0	1.0	1.0	1.0	1.0	0.0%	0.0%
	Test set	0.82	0.82	0.68	0.90	0.79	0.86	0.61	0.61	12.0%	6.0%
**Clostridium difficile (contigs)**	Training set	1.0	1.0	1.0	1.0	1.0	1.0	1.0	1.0	0.0%	0.0%
	Test set	0.95	0.95	0.93	0.97	0.95	0.97	0.90	0.90	3.5%	1.7%
**Klebsiella penumoniae (contigs)**	Training set	1.0	1.0	1.0	1.0	1.0	1.0	1.0	1.0	0.0%	0.0%
	Test set	0.79	0.75	0.94	0.22	0.58	0.97	0.23	0.20	4.8%	16.7%
**Klebsiella pneumoniae (reads)**	Training set	1.0	1.0	1.0	1.0	1.0	1.0	1.0	1.0	0.0%	0.0%
	Test set	0.86	0.86	0.91	0.67	0.79	0.98	0.58	0.58	7.1%	7.1%

Table M. Performance of linear regression model (N *k*-mers = 1,000)

Dataset		The mean squared error	The coefficient of determination (R^2)	The Pearson correlation coefficient and p-value	The Spearman correlation coefficient and p-value	The ±1 two-fold dilution factor accuracy
**Pseudomonas aeruginosa (contigs)**	Training set	2.21	0.77	0.88, 4.62E-50	0.88, 7.42E-50	0.71
	Test set	5.79	0.42	0.68, 5.31E-08	0.84, 2.48E-14	0.48
